# Effects of Anionic Liposome Delivery of All–*Trans*–Retinoic Acid on Neuroblastoma Cell Differentiation

**DOI:** 10.3390/biomimetics9050257

**Published:** 2024-04-24

**Authors:** Antonio Minò, Francesco Lopez, Roberto Barbaro, Maria Barile, Luigi Ambrosone, Matilde Colella

**Affiliations:** 1 Department of Biosciences and Territory (DiBT), University of Molise, Contrada Lappone, 86090 Pesche, Italy; antonio.mino@unimol.it; 2 Department of Medicine and Health Sciences “V. Tiberio”, University of Molise, Via F. De Sanctis snc, 86100 Campobasso, Italy; ambroson@unimol.it; 3 Department of Agricultural, Environmental and Food Sciences (DiAAA), University of Molise, Via F. De Sanctis snc, 86100 Campobasso, Italy; lopez@unimol.it; 4 Department of Biosciences, Biotechnology and Environment (DBBA), University of Bari “Aldo Moro”, Via Orabona 4, 70125 Bari, Italy; roberto.barbaro@uniba.it (R.B.); maria.barile@uniba.it (M.B.)

**Keywords:** all–*trans*–retinoic acid (ATRA), liposome, differentiation, neuroblastoma, morphological characterization, drug delivery, biomedical research

## Abstract

All–*trans*–retinoic acid (ATRA) has long been known to affect cell growth and differentiation. To improve ATRA’s therapeutic efficacy and pharmacodynamics, several delivery systems have been used. In this study, free ATRA and anionic–liposome–encapsulated ATRA were compared for their effects on SK–N–SH human neuroblastoma cell growth and differentiation. Anionic liposomes made of L–α–phosphatidylcholine (PC) and L–α–phosphatidic acid (PA), empty (PC–PA) and loaded with ATRA (PC–PA–ATRA), were characterized by dynamic light scattering (DLS) and electrophoretic mobility measurements, and drug entrapment efficiency (EE%) was measured to evaluate the applicability of the new colloidal formulation. The results of brightfield microscopy and cell growth curves indicated that ATRA, whether free or encapsulated, reduced growth and induced differentiation, resulting in SK–N–SH cells changing from epithelioid to neuronal–like morphologies, and producing a significant increase in neurite growth. To further characterize the neuro-differentiation of SK–N–SH cells, the expression of *β*III–Tubulin and synaptophysin and mitochondria localization were analyzed via immunofluorescence. Increased expression of neuronal markers and a peculiar localization of mitochondria in the neuritic extensions were apparent both in ATRA– and PC–PA–ATRA–differentiated cells. As a whole, our results strongly indicate that ATRA treatment, by any means, can induce the differentiation of parent SK–N–SH, and they highlight that its encapsulation in anionic liposomes increases its differentiation ability in terms of the percentage of neurite–bearing cells. Interestingly, our data also suggest an unexpected differentiation capability of anionic liposomes *per se*. This work highlights the importance of developing and carefully testing novel delivery nanocarriers, which are a necessary first “step” in the development of new therapeutic settings.

## 1. Introduction

Neuroblastoma is a pediatric tumor of the peripheral nervous system, which arises from multipotent neural crest–derived precursors and accounts for 15% of childhood cancer deaths [1]. Histologically, neuroblastoma is characterized by a variety of cellular phenotypes. This heterogeneity is maintained in the cell line used in this study, named SK–N–SH, which derives from bone marrow metastasis [2]. It has been more than four decades since retinoid (RAs) therapy was developed for neuroblastoma. The administration of RAs has been extensively demonstrated to result in cell differentiation and cell growth arrest [3].

The Food and Drug Administration (FDA) approved all–*trans*–retinoic acid (ATRA) as a treatment for acute promyelocytic leukemia (APL) in 1995. To date, it remains a central component of the treatment of APL in combination with other therapies [4,5,6,7]. As far as neuroblastoma therapy is concerned, several clinical trials have demonstrated retinoid effectiveness when used in combination with IFN–alpha [8] or intensive chemoradiotherapy, which significantly improved event–free survival in high–risk neuroblastoma [9]. A recent review by Makimoto and colleagues highlighted the clinical relevance of the use of isotretinoin (an endogenous retinoid agent that interconverts with ATRA) in neuroblastoma treatment [10].

The interest in retinoic acid for numerous additional biomedical applications has increased over the past ten years due to numerous discoveries about its biological role in regulating the biology of cancer resistance [11], cell reprogramming, and differentiation [12,13]. Over fifty active clinical trials have confirmed this interest [14].

Given retinoic acid’s clinical relevance and perspective, researchers are still looking for new ways to boost their effectiveness and decrease their numerous side effects [14,15]. When administered systemically, they interact with and activate a wide variety of signaling pathways in different tissues and organs [14]. Although extensively researched, the signaling pathways underlying retinoic acid effects remain an active area of research. Besides the well–known effects mediated by retinoid receptors (RXRs) and (RARs) [16,17], extranuclear and non–transcriptional effects of retinoic acid have also been demonstrated [18].

ATRA can trigger differentiation by controlling the activity of the ROR1 receptor tyrosine kinase–like orphan receptor (ROR1) [19]. Retinoids can also activate the mitogen-activated protein kinase (MAPK) pathway [20], regulate mitochondrial function [21], modulate microRNAs [22], and influence MYCN signaling [23].

Another important factor influencing ATRA’s usefulness in a therapeutic setting is its significant photo and chemical instability in the presence of oxygen and acids [24,25].

Retinoids’ encapsulation in various drug delivery systems is one known method of shielding them against destabilizing events, and liposomes are among the most adaptable carriers [26,27]. Recently, some of the authors of this paper demonstrated the ability of liposomes to encapsulate, protect, and deliver ATRA in an *in vitro* digestion process [28].

The use of liposomes in the administration of drugs has already had a significant influence in a variety of scientific fields [29]. Liposomes are a promising delivery system due to their physical and biochemical properties, which make them easy to manipulate [30,31,32,33]. It has been shown that liposomal preparations are useful carriers for stabilizing and protecting therapeutic drugs, enabling an efficient release of the encapsulated compounds at the targeted sites [34,35].

Numerous examples of ATRA encapsulated in liposomes can be found in the literature. Kitagawa et al. suggested the use of liposomes for the intradermal delivery of lipophilic drugs like retinoic acid [36]. ATRA–loaded liposomes have been used in the treatment of anaplastic thyroid carcinoma [37], and for *in vitro* and *in vivo* breast [38] and lung cancer studies [39,40].

Here, we aimed to unveil the capability of a novel anionic liposomal formulation to stabilize and protect the therapeutic compound ATRA and possibly increase its differentiation efficiency in an *in vitro* neuroblastoma model. For this purpose, we evaluated the growth, morphology, and neuronal marker expression of SK–N–SH neuroblastoma cells after short–term incubation (5–7 days) with ATRA encapsulated in anionic liposomes, as compared with free ATRA.

## 2. Materials and Methods

### 2.1. Materials

L–α–phosphatidylcholine (egg yolk lecithin) and L–α–phosphatidic acid (egg, chicken) (sodium salt) were purchased from Avanti Polar Lipids. RPMI–1640 w/L–glutamine was obtained from Euroclone (Pero, Italy); non–essential amino acids, trypsin, and penicillin/streptomycin were from Thermo Fisher Scientific, Waltham, MA, USA. SK–N–SH cells were purchased from IST (Istituto Scientifico Tumori of Genova, Italy). All other chemicals were purchased from Sigma–Aldrich (St. Louis, MO, USA) and used as received.

### 2.2. Liposome Preparation

Unilamellar anionic liposomes made of L–α–phosphatidylcholine (PC) and L–α–phosphatidic acid (PA) and ATRA were synthesized using the reversed–phase evaporation method as outlined by Szoka and Papahadjopoulos [41]. We dissolved 30 mg PC and 15 mg PA in 3 mL of diethyl ether then added 0.180 mL of ATRA dissolved in dimethyl sulfoxide (DMSO) (3 mg ${mL}^{-1}$). The organic phase was then combined with 1 mL of PBS buffer (pH 7.2) to form a two–phase system. This was achieved using a sonicator tip to produce an inverted micelle dispersion. Using a rotary evaporator, the organic solvent was eliminated, transforming the inverted micelles into an aqueous liposome solution. To eliminate any remaining solvent, two milliliters of buffer were added, and the suspension was then allowed to sit in the rotary evaporator for an additional 45 to 60 min. Before being used, the resulting multilamellar liposomes were successively extruded through 100 nm polycarbonate membranes.

### 2.3. Liposome Characterization

The average hydrodynamic diameter, the polydispersity index (PDI), and the ζ–potential values of the anionic liposome, empty or containing ATRA, were determined using dynamic light scattering (DLS) and electrophoretic mobility measurements at 25 °C using a Zetasizer–Nano ZS90 (Malvern, UK) commercial instrument powered by a 4 mW He–Ne laser (wavelength: 633 nm). At a fixed capillary cell level, laser Doppler electrophoresis measurements were performed. The scattered light was captured via forward scattering at a fixed detector angle of 17°. The distribution of electrophoretic mobility was found in the slow field inversion sequence, whereas the average was found in the rapid field inversion sequence. After that, the zeta potential was calculated using electrophoretic mobility according to the Smoluchowski method. DLS records were collected by leaving the instrument free to optimize the instrumental parameters. The hydrodynamic diameter size distribution was recovered by performing an inverse Laplace transform of the autocorrelation function (ACF) using software implemented by the manufacturer and then applying the Stokes–Einstein equation assuming the viscosity of the aqueous solution at 25 °C.

### 2.4. ATRA Entrapment Efficiency

The ATRA amount contained in the anionic liposomes was determined by spectrophotometric analysis, and its concentration was calculated from a calibration curve previously determined. The liposomal suspension containing ATRA was centrifuged at 4000 rpm for 10 min at room temperature. The supernatant and the pellet were divided and separately analyzed using a spectrophotometer (Cary Bio 100–Varian, Italy) at the ATRA $\lambda_{max}$ 365 nm. Before analysis, chloroform (3 mL) was added to the pellets for disruption. No interference deriving from the liposomal components was observed (an empty liposomal formulation was used as a blank). The ATRA amount entrapped in the liposomes was determined as the difference between the amount added during liposome preparation and the unentrapped value.

The encapsulation efficiency (EE%) of ATRA into anionic liposomes was expressed as the percentage of the total amount of ATRA that became entrapped, according to the following equation:

(1) $$EE\%=\frac{amount\;of\;ATRA\;entrapped\;in\;liposomes}{amount\;of\;ATRA\;loaded}\times 100$$

### 2.5. Cell Culture

SK–N–SH cells were cultured to a maximum of passage 10 from the original stock at 37 °C in a humidified atmosphere containing 5% ${CO}_{2}$/95% air in a complete medium composed of RPMI–1640 medium with L–glutamine, supplemented with 10% fetal bovine serum, 1 mM sodium pyruvate, 1% non–essential amino acids, and 1% penicillin/streptomycin. The maintenance culture was passaged once a week by gentle trypsinization. The cells were seeded on glass coverslips (at a density of 60–70%) and used 7 days after seeding for all the experiments.

### 2.6. Differentiation of SK–N–SH Cells and Liposome Treatments

At 24 h after seeding to a density of $2\times 10^{4}\;cells/{cm}^{2}$ on poly–L–lysine (100 μg/mL)–coated glass coverslips, the culture medium was removed and replaced with a fresh one containing 1 μM ATRA diluted in culture medium (final concentration of DMSO: <0.1%) and 1 μM ATRA encapsulated in anionic liposomes (PC–PA–ATRA 1 μM). In parallel, control wells were treated with fresh RPMI–1640 medium (CTRL) and empty anionic (PC–PA) liposomes diluted in culture medium. The differentiation media and the control group media were replaced at 48 h and 72 h. Cells were exposed to differentiation and control conditions for 7 days maximum.

### 2.7. Cell Growth Assessment

To determine growth curves, SK–N–SH cells were seeded at a confluence of $2\times 10^{4}\;cells/{cm}^{2}$ in a 12–well plate, previously treated with poly–L–lysine (100 μg/mL). At 24 h after seeding, cells were treated as described above with 1 μM ATRA and PC–PA–ATRA 1 μM. In parallel, control wells were treated with fresh RPMI–1640 medium (CTRL) and PC–PA empty liposomes diluted in culture medium. In each well, 10 randomly selected fields were captured using an inverted phase contrast Eclipse TE2000–S microscope (Nikon, Shinagawa, Tokyo, Japan) at 100× magnification, at 0, 1, 3, 5, and 7 days after treatment. Cells from each group (in triplicate) were counted using ImageJ software version 2.9.0/1.53t (National Institutes of Health, Bethesda, MD, USA).

### 2.8. Measurement of Neurite Outgrowth

The SK–N–SH cells ($2\times 10^{4}\;cells/{cm}^{2}$) were seeded in poly–L–lysine (100 μg/mL)–coated 12–well plates. At 24 h after seeding, cells treated for 7 days with the aforementioned treatments were tested for neurite growth at 0, 1, 3, 5, and 7 days after treatment. In each well, 10 randomly selected fields were captured (~100–200 cells) using an inverted phase contrast Eclipse TE2000–S microscope (Nikon, Shinagawa, Tokyo, Japan) at 100× magnification. Only the cells that exhibited extension of at least one neurite with a length longer than the diameter of the cell body were identified as neurite–bearing cells [19]. The neurite lengths of all neurite–bearing cells from each group (in triplicate) were measured using ImageJ software version 2.9.0/1.53t (National Institutes of Health, Bethesda, MD, USA) [42].

### 2.9. Cell Immunofluorescence and Epifluorescence Microscopy

Immunofluorescence protocols were previously described in Refs. [43,44]. Briefly, SK–N–SH cells seeded at $2\times 10^{4}cells/{cm}^{2}$ on poly–L–lysine (100 μg/mL)–coated 12 mm ∅ glass coverslips and subjected to the above–described treatments were stained with MitoTracker Red CMXRos (200 nM) for 30 min at 37 °C in serum–free RPMI. Following three PBS washes, cells were fixed with 3.7% paraformaldehyde/PBS for 20 min at RT and washed with PBS three times. Then, cells were permeabilized using Triton X–100 (0.1%) on a shaker for 15 min in the dark at RT and washed three times with PBS. Nuclei were stained with a Hoechst 33258/PBS solution (2 μM) for 15 min at RT in the dark. The coverslips were mounted on glass slides using 90% glycerol/PBS after three successive washing steps.

To visualize neuronal markers, cells were washed three times in phosphate–buffered saline (PBS) at room temperature and fixed with 4% formaldehyde/PBS for 20 min. Then, cells were permeabilized using Triton X–100 (0.1%) on a shaker for 15 min in the dark at RT and washed three times with PBS. Cells were blocked for 1 h at RT in 0.1% gelatin/PBS (blocking buffer) on a shaker in the dark. Incubation with anti–βIII–Tubulin (Sigma) and with anti–synaptophysin primary antibodies (1:100) was performed in a blocking buffer at 4 °C overnight in a humidified plate. After three 0.1% gelatin/PBS washes, cells were incubated with the secondary antibodies Alexa–Fluor–488 and Alexa–Fluor–555 (Thermo Fisher Scientific, Inc.) (diluted 1:500) in blocking buffer for 1 h at RT in a humidified plate. Cells were then washed with 0.1% gelatin/PBS three times and nuclei were stained with a Hoechst 33258/PBS solution (2 μM) for 15 min at RT in the dark. Epifluorescence microscopy at 100× magnification was performed using a Leica DMRXA microscope, connected to a Nikon DXm1200 digital camera equipped with the Leica Application Suite Advanced Fluorescence 3.1 image acquisition program. At least three independent staining experiments were performed. Representative images are reported in the figures.

### 2.10. Data Analysis

Data analysis was performed using GraphPad Prism (v. 4.00 GraphPad Software, Inc., San Diego, CA, USA). Analyses of variance that showed *p* < 0.05, *p* < 0.01, and *p* < 0.001 were followed by Bonferroni post hoc tests. The paired data were tested for statistical significance using the two–way ANOVA test. The data were expressed as means ± SE with *n* equal to the number of experimental runs.

## 3. Results

### 3.1. Physicochemical Characterization of Liposome–ATRA Formulations

The physicochemical properties of liposomal carriers are key in determining the final biopharmaceutical profile of the formulation. Small size and narrow size distribution are highly desirable, especially for systemically administered colloidal drug delivery systems.

As reported in Table 1, DLS analysis of our liposomal formulations showed no significant influence on the physicochemical properties of the liposomes following ATRA encapsulation.

The empty anionic liposomes (PC–PA) and the ATRA–loaded anionic liposomes (PC–PA–ATRA) showed a mean diameter of 124.0 nm and 150.0 nm, respectively, and a low polydispersity index (0.070 and 0.100). Furthermore, the surface charge of liposomes was not affected by ATRA encapsulation, consistent with previously reported findings [45]. The negative ζ–potential of the liposome formulations caused colloidal repulsion, preventing aggregation phenomena and ensuring colloidal stability.

Drug entrapment efficiency (EE%) is another important property that needs to be investigated to assess the applicability of colloidal formulations. The amount of ATRA entrapped within anionic liposomes was approximately 99% (0.6 mM) of the drug amount that was initially added. This result is due to the lipophilic character of ATRA, which induced the incorporation of this compound at the level of the liposome bilayer structure [28,46].

**Figure 1:**
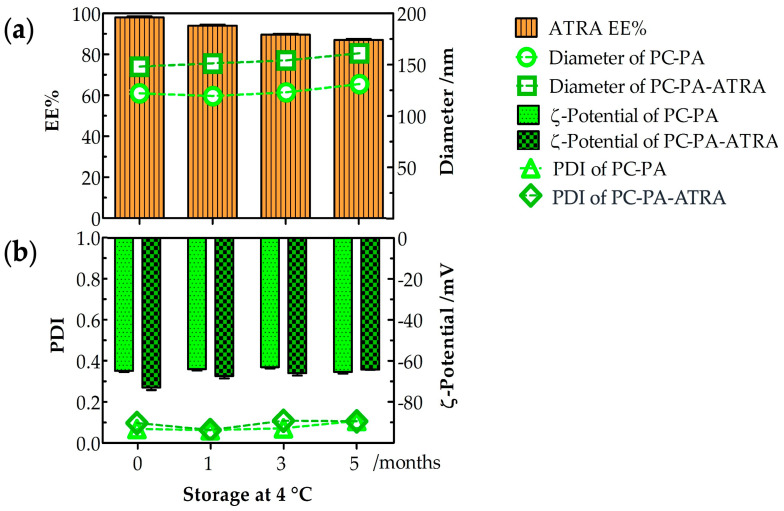
Empty and ATRA–loaded anionic liposome stability after 5–month storage at 4 °C (*n* = 3). (**a**) Entrapment efficiency (EE%), diameter, (**b**) PDI and ζ–potential of empty and ATRA–loaded anionic liposomes. PC: L–α–phosphatidylcholine; PA: L–α–phosphatidic acid; ATRA: all–*trans*–retinoic acid.

The physicochemical stability of empty and ATRA–loaded anionic liposomes was evaluated at 4 °C for 5 months, as shown in Figure 1.

The drug entrapment percentage indicates the storability of ATRA in anionic liposomes during a 5–month storage time, as shown in Figure 1a. The ATRA–loaded anionic liposomes retained their stability well at 4 °C. After 5 months at 4 °C, the formulation was highly stable, with 87% entrapment. Furthermore, after 5 months at 4 °C, the average diameter (Figure 1a) and PDI (Figure 1b) were measured. The average diameter was 130 nm (PDI: 0.110) for the PC–PA liposomes and 163 nm (PDI: 0.108) for the PC–PA–ATRA liposomes, while the ζ–potential (Figure 1b) was −65.3 mV for the PC–PA liposomes and −64.2 mV for PC–PA–ATRA liposomes. Therefore, no significant changes were observed in the physicochemical properties of empty and ATRA–loaded anionic liposomes during the stability study for formulations stored at 4 °C for 5 months. These results are in accordance with some studies performed previously. For example, Haghiralsadat et al. performed stability studies of liposomal doxorubicin, stored at 4 °C for 6 months, demonstrating that the liposomal formulation minimizes problems associated with liposome instability [47]. Furthermore, Said et al. studied the physicochemical properties of colloidal nanosystems containing retinoic acid after storage at 4 °C for the duration of the study (6 months).

No apparent aggregation or precipitation occurred in our liposomal formulations during storage time, nor did it occur for their colloidal nanosystems. Their physicochemical features (particle size, PDI, ζ–potential, and EE%) remained unchanged, indicating that they retained their colloidal properties [48].

### 3.2. Morphological Analysis and Cell Growth Assessment

**Figure 2:**
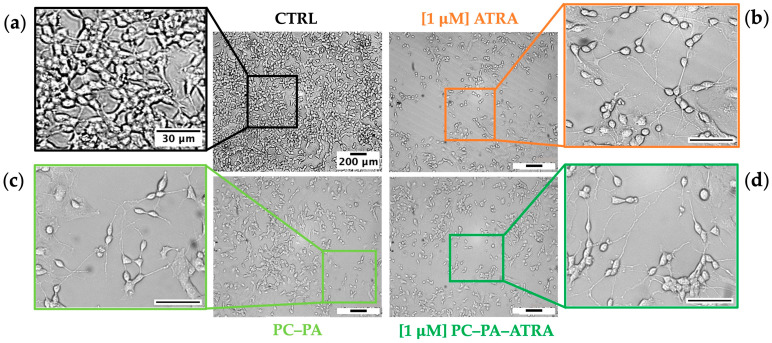
Brightfield images of SK–N–SH cells showing the typical cell morphology of (**a**) untreated control cells (CTRL) and cells incubated for 5 days with (**b**) 1 μM ATRA, (**c**) PC–PA liposomes, and (**d**) 1 μM PC–PA–ATRA liposomes. Outgrowths of neurites, neurite branching, and the development of growth cones were observable in (**b**–**d**). Scale bars of the original micrographs (magnification 100×) and insets denote 200 μm and 30 μm lengths, respectively. PC: L–α–phosphatidylcholine; PA: L–α–phosphatidic acid; ATRA: all–*trans*–retinoic acid.

To assess the effects of 1 μM free– and encapsulated–ATRA on SK–N–SH morphological differentiation, 10 randomly selected brightfield images were captured in at least three wells per condition after 5 days of incubation. As apparent from the representative images shown in Figure 2, control samples (Figure 2a) mainly consisted of cells with an undefined cell phenotype, displaying either rounded or fibroblastic morphology with a quadrangular cell shape. These cells, characterized by a high proliferation rate, also showed a clear tendency to form clusters. Only a few short neurites were observable in control samples in comparison with cells treated with 1 μM ATRA (Figure 2b). Here, as expected from previous studies [49,50], a notable reduction in the total number of cells and a prevalence of cells with a globose cell body with numerous neurites was evident.

Figure 2c shows SK–N–SH cells treated with empty PC–PA anionic liposomes. Unexpectedly, a consistent increase in differentiated cells, as demonstrated by the considerable increase of neurites compared to the control, is apparent. A corresponding growth inhibition compared to control was also observed in these samples. Significantly, samples treated with liposomes loaded with ATRA also showed a marked inhibition of growth in addition to an increase in differentiation (Figure 2d), as highlighted by the strong reduction in the number of cells compared to proliferating control cells, as well as by the increased number of neuritic extensions.

In parallel, to obtain semiquantitative information on the effects of the different treatments on SK–N–SH cell differentiation, growth curves were plotted.

**Figure 3:**
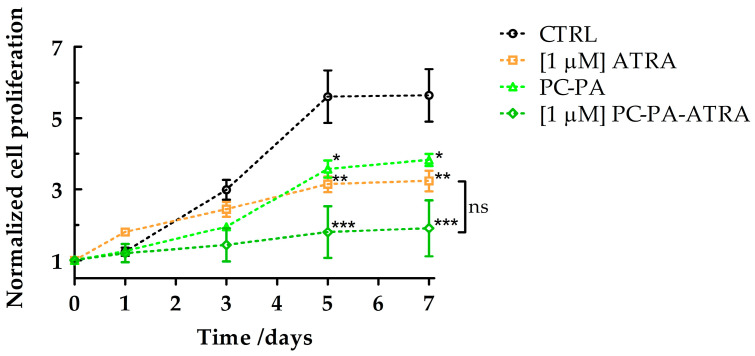
Growth curves of SK–N–SH cells treated with 1 μM ATRA, PC–PA, and 1 μM PC–PA–ATRA liposomes. Growth curves are normalized to the number of cells counted at time 0 for each treatment. Data were subjected to analyses of variance followed by multiple pair–comparison post-hoc tests using Bonferroni’s correction (* = *p* value < 0.05; ** = *p* value < 0.01; *** = *p* value < 0.001 vs. control) (*n* = 3). PC: L–α–phosphatidylcholine; PA: L–α–phosphatidic acid; ATRA: all–*trans*–retinoic acid.

The results shown in Figure 3 confirmed the ability of all the treatments containing 1 μM ATRA, either free or encapsulated, to reduce cell growth. Again, this analysis revealed a notable reduction in the number of cells (suggestive of differentiation given the morphological data, Figure 2) also in the samples treated with empty anionic liposomes, which performed similarly to ATRA treatment. It has to be noted that, at the beginning of the work, the impact of modified nanoliposomes on cytotoxicity was verified using the Trypan blue dye assay [51]. Importantly, in our experimental conditions, no difference in the percentage of trypan blue positive cells was measurable on the fifth day of incubation with ATRA, either free or embedded in liposomes, compared with the control. Similarly, empty liposomes did not exert any cytotoxic effects.

**Figure 4:**
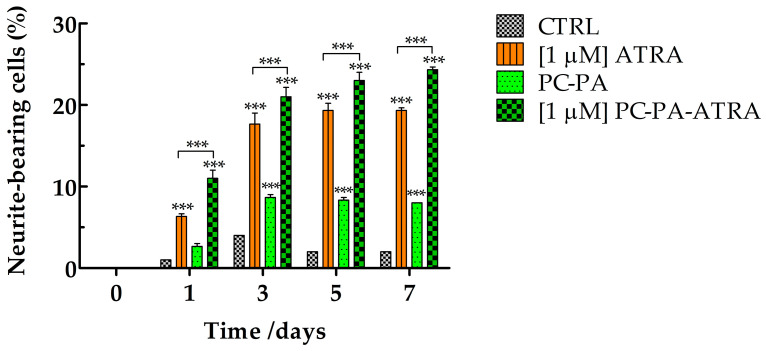
Percentage of neurite–bearing SK–N–SH cells in control conditions and after treatment with 1 μM ATRA, PC–PA, and 1 μM PC–PA–ATRA liposomes. The percentages of cells bearing at least one neurite with a length longer than the diameter of the cell body were calculated. Data are expressed as the percentage of cells bearing at least one neurite over total cells (180 to 200 cells). *** *p* < 0.001 vs. control cells and cells treated with 1 μM ATRA (*n* = 3). PC: L–α–phosphatidylcholine; PA: L–α–phosphatidic acid; ATRA: all–*trans*–retinoic acid.

Importantly, a semiquantitative analysis confirmed that encapsulated ATRA induced a significant increase in the number of neurite–bearing cells compared to the control and ATRA–treated cells (Figure 4). To a lesser extent, empty liposomes also induced a significant increase in the percentage of neurite-bearing cells compared to the control.

### 3.3. Immunofluorescence Assays

To specifically evaluate the expression of neuronal markers and obtain more details on the morphological characteristics of differentiated SK–N–SH cells, immunofluorescence studies were performed in all experimental conditions.

**Figure 5:**
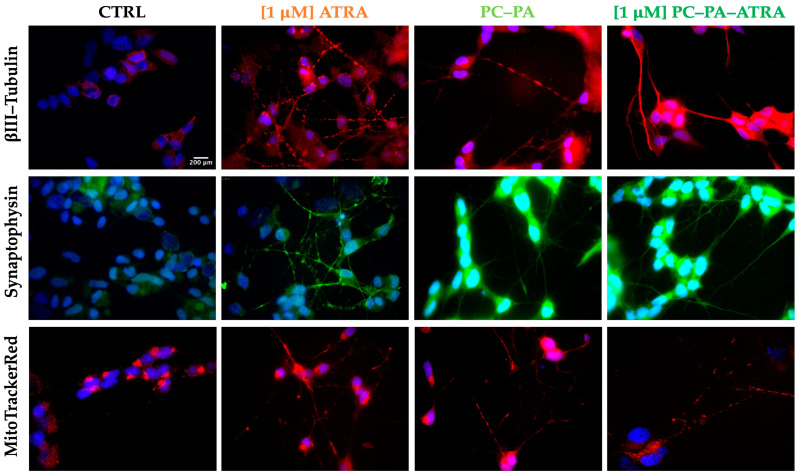
Immunofluorescence characterization of control and treated SK–N–SH cells. From left to right, representative fluorescence–merged microphotographs of control cells and cells treated with 1 μM ATRA, PC–PA liposomes, and 1 μM PC–PA–ATRA liposomes for 6 days. The rows show *β*III–Tubulin–positive (red fluorescence, first row), synaptophysin–positive (green fluorescence, second row) and MitoTracker CMX ROS stained cells (red, third row). Nuclei were stained with Hoechst 33258. Scale bar: 200 μm (100×). PC: L–α–phosphatidylcholine; PA: L–α–phosphatidic acid; ATRA: all–*trans*–retinoic acid.

First, the subcellular localization of βIII–Tubulin, broadly used as a marker of neuronal identity and differentiation, was assessed. After fixation, permeabilization, and incubation with the anti– βIII–Tubulin-specific antibody, the immunocomplexes were visualized with a secondary antibody conjugated with Alexa Fluor 568 (red signal). Nuclei were counterstained with Hoechst 33258 (blue signal). The representative merged images are shown in the first row of Figure 5.

A high–intensity red signal of βIII–Tubulin was apparent in all the differentiated cells with highly concentrated immunocomplexes in neurites. In contrast, untreated control cells displayed a dimmer red signal with an intensity comparable to the range of background fluorescence, mainly localized on cell soma.

Next, immunolocalization of synaptophysin, a classical neuronal marker, was revealed (second row of Figure 5). Again, in all the treated cells, expression of the neuronal marker synaptophysin was apparent as a clear sharp green signal, highlighting the neurites and neurite branching. Control cells showed a dim signal mostly present around the nuclei.

In a subsequent experiment, MitoTracker CMX Ros (200 nM) was used for mitochondria visualization (Figure 5). In this case, the cells were loaded with the fluorophore for 20 min in the incubator and thereafter subjected to fixation, permeabilization, and mounting procedures. As evident from the images in Figure 5, this assay revealed that, as expected in functionally active neurons, mitochondria were distributed and sometimes enriched at the neurite terminals of differentiated cells, while they appeared curled up at one pole of the cell, near the nucleus, in parental SK–N–SH cells. In the differentiation process, a “migration” of the mitochondria in the neuritic extensions is thus observed, indicating an increase in mitochondrial transport to the sites of synthesis and release of neurotransmitters, which require high energy expenditure.

As a whole, the representative micrographs depicted in Figure 5 clearly demonstrate that cells differentiated with 1 μM ATRA, free or encapsulated in anionic liposomes, emit long neurites and express a high degree of classical markers of neural differentiation, ultimately confirming the observation obtained in brightfield (Figure 2). This evidence, along with the observation of mitochondria localized in neurites, strongly suggests a functional polarization of cells that seem to specialize for neurotransmission (axonal transport of mitochondria, accumulation of synaptic vesicles). Furthermore, immunofluorescence data confirmed the unexpected differentiating effect of empty anionic PC–PA liposomes.

## 4. Discussion

Our results demonstrate that the encapsulation of ATRA in PC–PA anionic liposomes, at the very least, preserves its differentiation ability as measured by morphological differentiation and antiproliferative effect while significantly increasing neurite outgrowth.

Also, our study uncovered an unexpected differentiating effect of empty PC–PA liposomes. Although a number of speculations could be made to explain such an observation, starting from the effect of lipids on plasma membrane channels [52] or enzymes [53] to arrive at their effect on nuclear receptors [54], further mechanistic studies are needed to uncover the signaling pathways causative of such unexpected but straightforward observations.

The objective of this study is to develop and characterize a novel liposomal carrier for ATRA. On the one hand, the novel PC–PA anionic formulation could provide insight into the general properties of nanocarriers; on the other hand, after “*in vivo*” testing, it could ameliorate the field of ATRA’s use in clinics through the improvement of its cellular absorption and bioavailability.

As stated above, the clinical efficacy of ATRA in cancer treatment is hindered by a number of drawbacks, including its short biological half–life in humans [55] and its hydrophobicity and sensitivity to light, heat, and oxidants [15].

To enhance ATRA’s efficacy, researchers are primarily concentrating on two areas: (i) evaluating the combination with different anti–tumor agents to decrease ATRA concentration and thus overcome drug resistance [56], or (ii) enhancing its delivery efficacy by using drug carriers. An excellent example of this is liposomes, which, if designed appropriately, can enhance bioavailability, reducing the toxicity of high doses and the non–specificity of ATRA [57].

There are three fundamental types of liposomes: cationic, anionic, and neutral. During synthesis, their composition, charge, and size can be changed to fulfil stability, bioavailability, and therapeutic efficacy objectives [58]. There are usually advantages and downsides to each type of liposome [27].

Although anionic liposomes are often less inclined to interact with biological membranes due to their equally negative charge compared to cationic ones, this trait also reduces the possibility that the immune system will target them as foreign bodies [59]. This has been observed with cationic liposomes, which exhibit, despite a higher uptake rate [60], poor activity due to antibody coating and phagocyte ingestion, and higher cytotoxicity [61]. Anionic liposomes have been widely used for drug delivery [62], and one formulation for treating ovarian and breast cancer has been approved by the FDA [27].

## 5. Conclusions and Future Perspectives

In light of our results, it has become increasingly clear that the development and careful characterization of novel liposomal formulations is of paramount importance for the development of more efficient drug delivery strategies. In the case of ATRA, a more efficient cell differentiation effect would be an essential goal for both the clinics and the biomedical research community. Also, more effective differentiation methods would allow the optimization of *in vitro* neuronal experimental models essential in pharmacological, biochemical, and cell physiology assays, all crucial for the dissection of the molecular mechanisms involved in the pathogenesis of neuronal diseases.

To confirm the stability and high encapsulation efficiency of PC–PA–ATRA liposomes, it would be worthwhile to investigate these properties in animal models. This would support the evaluation of these liposomes as therapeutic agents.

Further, the development of specifically targeted or stimuli–responsive ATRA–loaded liposomes [63,64] would represent a fundamental advancement in this field.

## Figures and Tables

**Table 1:** Liposome parameters.

** 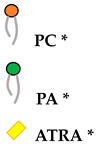 **	** 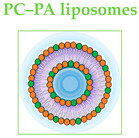 **	** 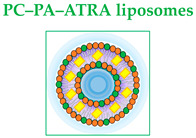 **
**Diameter/nm**	124.0 ± 0.5	150.0 ± 0.3
**PDI**	0.070 ± 0.007	0.100 ± 0.011
ζ **–potential/mV**	−64.8 ± 13.3	−72.9 ± 12.2
**EE%**		99%

* PC: L–α–phosphatidylcholine; PA: L–α–phosphatidic acid; ATRA: all–*trans–*retinoic acid.

## Data Availability

The raw data supporting the conclusions of this article will be made available by the authors on request.
